# Microstructural Evolution in a 6060 Extrudable Al-Alloy: Integrated Modeling and Experimental Validation

**DOI:** 10.3390/ma17030545

**Published:** 2024-01-23

**Authors:** John S. Aristeidakis, Gregory N. Haidemenopoulos, Ruben Bjørge, Calin D. Marioara, Helen Kamoutsi, Evangelos Giarmas, Nikolaos Rafailidis

**Affiliations:** 1 Department of Mechanical Engineering, University of Thessaly, 38334 Volos, Greece; ekamoutsi@o365.uth.gr; 2 Department of Materials and Nanotechnology, SINTEF Industry, 7465 Trondheim, Norway; ruben.bjorge@sintef.no (R.B.); calin.d.marioara@sintef.no (C.D.M.); 3 Alumil Aluminium Industry S.A., 61100 Kilkis, Greece; v.giarmas@alumil.com (E.G.); n.rafailidis@alumil.com (N.R.)

**Keywords:** extrudable Al-alloys, ICME, modeling, characterization, casting, homogenization, aging

## Abstract

Desirable properties including strength, ductility and extrudability of 6060 Al-alloys are highly dependent on processing to control the development of microstructural features. In this study, the process chain of an extrudable 6060 Al-alloy was modeled in an Integrated Computational Materials Engineering framework and validated experimentally via quantitative SEM-EDX and TEM. All critical processing stages were considered including casting, homogenization heating and holding, extrusion cooling and two-stage aging. Segregation and intermetallics formation were accurately predicted and experimentally verified in the as-cast condition. Diffusion simulations predicted the dissolution of intermetallics and completion of β-AlFeSi to α-AlFeSi transformation during homogenization, in excellent agreement with quantitative SEM-EDX characterization. Precipitation simulations predicted the development of a β″ strengthening dispersion during extrusion cooling and aging. Needle-shaped β″ precipitates were observed and analyzed with quantitative high-resolution TEM, validating predictions. Ensuing precipitation strengthening was modeled in terms of aging time, presenting good agreement with yield strength measurements. Precipitate-Free Zones and coarse, metastable β-type particles on dispersoids and grain boundaries were investigated. The proposed integrated modeling and characterization approach considers all critical processing stages and could be used to optimize processing of extrudable 6xxx Al-alloys, providing insight to mechanisms controlling microstructural evolution and resulting properties.

## 1. Introduction

The development of optimized microstructures and properties of extruded profiles of 6xxx alloys depends on the full understanding and control of the individual elements of the process chain. The design of these alloys depends entirely on the development of reliable and experimentally validated models and simulation tools to describe the microstructural evolution during processing. The 6xxx extrudable Al-alloys have a process chain comprising of casting in billets (solidification), homogenization of the billets, extrusion, and aging. Coarse Fe-rich intermetallics, eutectics and elemental segregation at grain boundaries and secondary dendrite arms that form upon solidification limit extrudability [1,2]. The β-AlFeSi phase in particular can be very detrimental for extrusion, being prone to nucleating cracks due to its monoclinic structure and plate morphology [3]. Homogenization aims at eliminating the as-cast structure and transforming the β-AlFeSi to α-AlFeSi phase with a cubic structure and globular morphology which is less impactful to extrudability [4,5,6]. Extrusion is followed by artificial aging to precipitate a fine dispersion of a strengthening phase, typically the metastable β″ in 6060 Al-alloys [7]. Extrusion cooling and aging conditions determine the phase fraction and size distribution of precipitates and therefore control the ensuing mechanical properties. In particular, extrusion cooling affects the formation of Precipitate-Free Zones which can limit ductility [8].

Modeling efforts in the past have mainly focused on describing individual elements of the process chain. The present work is an effort to integrate the simulation of solidification, homogenization, cooling after extrusion and artificial aging where the results of each process step are used as initial conditions for the next processing step, with experimental validation of simulation results at all process steps.

Many authors have developed intricate models and characterization techniques to study particular microstructural features or individual aspects of the process chain. Starting from solidification during ingot casting, Du et al. [9] employed a numerical micromodel approach for the prediction of intermetallic phase formation and microsegregation in A356 alloy and compared the model with equilibrium and Scheil-Gulliver solidification calculations. The model incorporated solid back-diffusion, dendrite tip and eutectic undercooling and used an optimized thermodynamic database description for the Al-Mg-Si-Fe-Mn system. They noticed discrepancies when comparing solidification paths and microsegregation between equilibrium and Scheil-Gulliver calculations with experimental data. However, a better agreement was obtained when the micromodel was used. Cinkilic et al. [10] investigated the effect of Mn on the formation of iron intermetallics during solidification using CALPHAD-based modeling. They established a relationship between Fe/Mn ratio and cooling rate to minimize the formation of the detrimental β-AlFeSi phase in the as-cast microstructure in Al-Mg-Si casting alloys. Both above works were on Al-Mg-Si-based casting alloys. Regarding extrudable alloys, Sarafoglou and Haidemenopoulos [11] employed CALPHAD-based modeling and provided maps, in the Mg-Si composition space, of phase fractions of Mg_2_Si and iron intermetallics for a large composition range (0–1.2 wt.% Mg and Si) in 6xxx alloys. The calculations were validated experimentally.

Regarding microstructure evolution during homogenization, several modeling approaches have been employed. A finite element approach was developed by Kuijpers et al. [12], to describe the β-AlFeSi to α-AlFeSi transformation and investigate the influence of several process parameters. The model was used in another work in [13] to study the effect of Si and Mn on the transformation kinetics and validated experimentally for rather short homogenization times. The interaction between Si and Mn were further investigated by Du et al. [14], employing a diffusion model. They also presented a coupled CALPHAD-based and Kampmann-Wagner-Numerical (KWN) model to predict the competitive nucleation and growth of precipitates during homogenization cooling in a 6082 alloy. They found that a multimodal Mg_2_Si particle size distribution develops during homogenization cooling. A diffusion model was also developed by Priya et al. [15], to study dissolution during homogenization holding as well as precipitation during homogenization cooling. Finally, Sarafoglou et al. [5] developed a CALPHAD-based dual-grain model to consider the inhomogeneous grain size on homogenization holding and homogenization cooling. The precipitation model they also developed was based on the KWN approach and was coupled with a strength model to predict the yield strength of the as-homogenized billets. In addition to homogenization cooling, non-isothermal precipitation reactions also take place during cooling following the extrusion process, often called quench sensitivity. A physically-based model of precipitation coupled with a strength model was introduced by Milkereit and Starink [16] to account for the quench sensitivity in several Al-Mg-Si alloys at a range of cooling rates. Precipitation during the isothermal aging treatment has been addressed by several researchers. Myhr et al. [17] developed a model based on the work presented in [18,19] to describe precipitation during thermal processing in a 6005, 6060 and 6063 Al-alloys with good agreement with experimental results. Du et al. [20] developed a model, based on the KWN framework, to predict precipitation during aging of Al-Sc and Al-Sc-Zr alloys. The work highlighted the sensitivity of the model to thermodynamic description of phases, interfacial energy and diffusivity of alloying elements. The model was further applied to Al-Mg-Si alloys in [21] with emphasis on the overaging of the needle-shaped β″ precipitates. A multiscale model based on Density Functional Theory (DFT), Monte-Carlo (MC) and phase-field approaches was developed by Kleiven and Akola [22], aiming at investigating the formation of Mg-Si rich precipitates in the FCC matrix of Al-Mg-Si alloys, showing that the precipitates tend to form elongated domains either by Ostwald ripening or by coalescence, as precursors to the needle-like β″ precipitates. Finally, Baganis et al. [23] presented a phase field simulation of precipitation in 6061 and 6082 alloys predicting under-aging and peak-aging conditions for different aging temperatures.

Despite the extensive work on the field in recent years, most prior studies have focused on individual process steps. In the present work, an integrated simulation and characterization study is provided. Emphasis is placed on modeling key microstructural features throughout processing, including (a) microsegregation and phase formation during solidification, (b) phase transformations and removal of microsegregation during homogenization heating and holding, (c) precipitation during cooling after extrusion and (d) precipitation and associated strengthening during artificial aging. Individual processing steps are linked, utilizing microstructural simulation results as initial conditions to the next one, with in-depth characterization at critical stages of processing providing validation of numerical predictions. Combining microstructural modeling and characterization efforts provides insight into the underlying mechanisms controlling the development of microstructural features and ensuing properties upon processing of extrudable Al alloys. The novelty of the present study lies in modeling and experimental investigation of key microstructural features during processing, that control the final properties of extrudable Al alloys. The developed approach demonstrates that by utilizing targeted experiments for calibration and validation of models, it is possible to predict the influence of processing conditions on microstructural features and ensuing material properties. Calibrated model predictions can then be employed to assess the influence of process modifications, minimizing trial-and-error experiments required to optimize industrial processes.

## 2. Materials and Methods

CALPHAD-based simulation of microstructure evolution was employed in the present paper, including the following aspects of the process chain: evolution of phase fractions and microsegregation during solidification and solidification cooling, evolution of phase fractions and compositions during homogenization and homogenization cooling, precipitation of intermetallic phases during extrusion cooling and artificial aging. Thermodynamic calculations were carried out in Thermo-Calc (Thermo-Calc Software AB, Solna, Sweden) version 2021b [24], diffusion calculations in DICTRA (v. 2021b) [25] and precipitation simulations in the Thermo-Calc precipitation module TC-PRISMA (v. 2021b), with the TCAL8 thermodynamic and MOBAL7 mobility database for aluminum alloys [26], in accordance with previously published work by the authors [5,11]. Extended experimental validation of model predictions was carried out at all process steps, using optical microscopy, SEM-EDX and TEM analysis as described below.

### 2.1. Simulation Methodology

#### 2.1.1. Solidification

Solidification of the material in a billet form was studied via the Scheil-Gulliver model in accordance with the work presented in [5,11]. The classic Scheil model as implemented in Thermo-Calc and coupled with the TCAL8 thermodynamic database was used in this study. The Scheil equation [27] predicts the solute redistribution during solidification, assuming that the solidification rate is high enough such that diffusion in the solid phases is negligible, diffusion in the liquid is infinite and local equilibrium conditions are established on the solid-liquid interface. The classic Scheil model is independent of the cooling rate, however modifications to account for back diffusion when cooling rates are low, or solute trapping at high cooling rates have been developed [27,28]. The approach has been validated experimentally in a variety of cases, including casting [5,11] and additive manufacturing [29] providing reasonable accuracy. In the present study, the classic calculation was used to predict elemental microsegregation development between primary and secondary dendrites, non-equilibrium intermetallics and eutectic mixture formation, as well as quantification of freezing ranges, upon casting with moderate cooling rates. All major alloying elements and phases were considered, including Mg, Si, Fe and Mn, and phases such as α-Al (FCC) matrix, β-Mg_2_Si, β-AlFeSi (Al_9_Fe_2_Si_2_), α-AlFeSi (Al_8_Fe_2_Si), π-phase (Al_18_Fe_2_Mg_7_Si_10_) and diamond (Si). The selection of phases was based on experimental evidence from the literature for 6xxx Al alloys [5].

#### 2.1.2. Solidification Cooling

The temperature profile on cooling upon casting in billets was considered by employing one-dimensional radial heat transfer simulations. Temperature-dependent thermophysical data required for the simulation were calculated according to CALPHAD-based equilibrium thermodynamics in Thermo-Calc, with the chemical composition of a 6060 Al-alloy. A custom finite difference approximation code was implemented to solve the one-dimensional heat transfer equations in cylindrical geometries, with temperature-dependent thermophysical properties. During casting, the outer surface of the billet remains in contact with water, therefore the temperature stays approximately equal to 20 °C, setting the boundary condition for the outer billet surface. Leveraging the radial symmetry of the billet, a zero-flux boundary condition was enforced at the center of the billet. The solution of the radial heat transfer equations determines the evolution of temperature profiles along the radius of the billet upon cooling. Calculated temperature profiles in addition to Scheil segregation profiles were then provided as input for subsequent DICTRA diffusion simulations to determine the microstructural evolution during solidification cooling, as elaborated in the following section.

#### 2.1.3. Homogenization

Diffusion simulations in multi-component systems were performed in DICTRA to predict the evolution of microstructural features such as segregation profiles and intermetallic phases during solidification cooling, homogenization heating and holding. The temperature profiles form heat transfer simulations were used for solidification cooling. For subsequent homogenization simulations, a typical heating cycle for batch homogenization of 6060 Al-alloys was used, during which the material is gradually heated and held to a temperature up to 580 °C over the course of several hours. The elimination of elemental segregation in the interdendritic space, the transformation of β-AlFeSi to the more favorable α-AlFeSi and the dissolution of undesired intermetallic compounds and mixtures [30] was studied. The segregation profile and intermetallic phase fractions provided by Scheil-Gulliver solidification simulations were used as initial conditions. Mass transfer equations were solved in DICTRA in a planar geometry using a 50 μm diffusion cell with an FCC matrix structure, representative of the primary dendritic arm spacing (half the diameter of the grain). Intermetallics forming at late stages of solidification were treated as dispersed phases in the FCC matrix and their contribution to the total local diffusional mobilities was considered via a “Rule of Mixtures” homogenization function. The modeling approach has been described in detail in [5], for a diffusion couple considering two grains with different diameters. In this study, a simpler geometry is considered but with a more accurate time-temperature profile. Results from the homogenization simulations can then be the starting point for additional precipitation and strengthening calculations, to model the aging process, after extrusion.

#### 2.1.4. Extrusion Cooling and Artificial Aging

Precipitation of fine metastable β″ particles during extrusion cooling and subsequent aging was simulated via computational alloy thermodynamics and kinetics modeling. Nucleation, growth and coarsening of precipitates during processing were calculated according to the Kampmann-Wagner (KWN) model [5,31], coupled with CALPHAD-based thermodynamic calculations in TC-PRISMA. The approach allows for the determination of the particle size distribution (PSD), average radius and needle length, number density and volume fraction of precipitates during processing.

For precipitation calculations, the complete thermal cycle of the material after extrusion was considered based on furnace temperature measurements, including extrusion cooling, and double aging. Linear cooling from the maximum extrusion temperature to ambient was considered, followed by linear heating and holding at a low aging temperature $T_{Ag}^{L}$. A second linear heating stage, isothermal holding at the high aging temperature $T_{Ag}^{H}$ and linear cooling to ambient follows to complete the heat treatment. Both aging temperatures were within typical aging temperature ranges for 6060Al, with $T_{Ag}^{L}$ measured between 165 and 180 °C and $T_{Ag}^{L}$ between 195 and 210 °C. The short time between extrusion cooling and aging, during which the material might remain at ambient temperature and be subjected to natural aging was also investigated, concluding that the effect is negligible for short times and thus omitted in the final calculation.

During preheating of the billet before extrusion, most of the β-family precipitates (i.e., β-Mg_2_Si, β″, β′, U1, U2 and B′) that might have formed during homogenization cooling, are dissolved in the matrix. Upon extrusion, the billet is adiabatically heated due to deformation and the temperature rapidly rises. It is reasonable to assume that by the time the profile exits the extrusion die, all β-family particles are dissolved and only coarse α-AlFeSi and dispersoids remain. Since holding at the maximum extrusion temperature is short, equilibrium conditions are not established at that temperature, however since all β-family particles are dissolved, the conditions closely approximate the conditions at the end of homogenization and prior to cooling, which can be used as the initial conditions for subsequent precipitation calculations.

The precipitation of a single phase of β″-Mg_5_Al_2_Si_4_ during extrusion cooling and aging was considered, as it is the major strengthening precipitate after aging, as suggested by the TEM analysis described later in the paper. The formation of overaging β-type precipitates such as β′, U1, U2 and B′, on grain boundaries and dispersoids during extrusion cooling was neglected and the β″-Mg_5_Al_2_Si_4_ needles were modeled as prolate spheroids with a constant aspect ratio, in the interest of simplicity. In reality the aspect ratio of β″ changes during aging, with the early needles being thick and short. The length then increases at a higher rate than the cross-section, so the aspect ratio increases during aging to minimize the surface energy of the precipitate since the top and bottom surfaces are incoherent. Yet, as observed by TEM, the sides of β″ needles remained fully coherent with the matrix. As a result, the interfacial energy is relatively low, and the contribution of the elastic strain energy is significant due to the development of coherency strains. Both effects were considered in TC-Prisma, with the elastic strain energy calculated according to the volumetric difference, and the interfacial energy according to CALPHAD, as a function of composition and temperature. According to the literature, the interfacial energy of β″, varies from 100 to 300 mJ/m^2^ [32,33,34] depending on the crystallographic orientation of the interface. In the present study, the interfacial energy model was adjusted using a prefactor to result in a value of 110 mJ/m^2^, considering that β″ is optimally oriented along the <100> direction. Additionally, heterogeneous nucleation on vacancy clusters in the bulk of the material was considered, with a number of nucleation sites per unit volume equal to 8 × 10^28^ m^−3^.

In the TC-Prisma precipitation model, needle particles are represented as prolate ellipsoids with a small and a large radius, whereas in reality β″ particles have a cylindrical geometry with a rhomboidal cross-section. The small ellipsoid radius in the computational geometry corresponds to the large radius of the β″ rhombus cross-section in the physical geometry. If the rhomboidal cross-section of β″ has a side α and an acute angle θ = 75° (oblique angle 105°) from the monoclinic unit cell of β″, it can be shown that the large rhombus diagonal is $D_{rh}=\alpha \sqrt{2+2\cos\left(\theta \right)}$. The cross-sectional area can then be calculated as $A=\frac{1+\cos\left(\theta \right)}{2\;sin(\theta )}r^{2}≅1.5374r^{2}$, where $r=D_{rh}/2$ is the large rhombus radius corresponding also to the calculated small ellipsoid radius. The expression can then be used to compare cross-sectional area measurements with model predictions as discussed in Section 3.2.5. Similarly, the large ellipsoid radius in the computational geometry corresponds to half the β″ needle length in the physical geometry, with a conversion factor such that the particle volume is maintained. Comparing the predicted and measured needle length requires the application of a correction factor to convert between the two geometries. Considering that the particle volume between the two geometries should be maintained constant we obtain $V_{e}=V_{r}\Rightarrow \frac{4}{3}\pi r_{e}^{2}\frac{L_{e}}{2}=1.5374r_{r}^{2}L_{r}$, where $L_{e}$ is the ellipsoid length, i.e., twice the large ellipsoid diameter and $L_{r}$ is the physical length of rhomboidal β″ needles. Equating the radii then gives the conversion factor between lengths in the two geometries as $L_{r}≅1.3647L_{e}$. The ellipsoid length $L_{e}$ is computed from TC-Prisma precipitation simulations and then $L_{r}$ can be compared with the measured length of β″ needles. Note that in all calculation results presented in the following sections, the length correction has been applied.

#### 2.1.5. Strengthening

Evolution of yield strength with aging time was calculated based on β″ distribution predictions in TC-PRISMA according to the yield strength property model of Thermo-Calc. Even though Thermo-Calc’s proprietary model was used in this study, the basic equations are well-established in the literature and have also been presented in detail in previous work by the authors of [5,35,36]. More specifically, the total room temperature yield strength was calculated in terms of the solid solution, grain boundary, dislocation forest and precipitation strengthening contributions [5,35,36]. Solid solution strengthening includes the intrinsic resistance of the aluminum matrix to deformation, along with the additional strengthening from solute atoms. The grain boundary strengthening mechanism originates from the interaction of dislocations with grain boundaries, increasing as the grain size becomes smaller. The dislocation forest strengthening contribution arises from the interaction of dislocations with other dislocations, becoming more prominent when the dislocation density increases, e.g., during plastic deformation. In the present study, the grain boundary and dislocation forest strengthening contributions to the macroscopic yield stress were considered constant throughout extrusion cooling and aging.

Precipitation strengthening is the dominant hardening mechanism after aging of 6060Al, arising from the interaction of dislocations with very fine β″ precipitates. In small, coherent particles, coherency hardening is the primary precipitation strengthening mechanism, as dislocations encounter resistance as they glide through the particle [35]. Coherency strengthening $\sigma_{p}^{coh}$ becomes more prominent when the mean radius $\bar{r}$ and the volume fraction f of the shearable particles increases, with $\sigma_{p}^{coh}\sim \sqrt{f}\sqrt{\bar{r}}$. In large or not fully coherent particles, the main precipitation strengthening mechanism is Orowan strengthening, during which dislocations loop around unshearable precipitates and their movement is inhibited. Orowan strengthening $\sigma_{p}^{Orow}$ is more prominent when for a given volume fraction, the interparticle distance becomes smaller, i.e., when the precipitate size decreases, with $\sigma_{p}^{Orow}\sim \sqrt{f}/\bar{r}$. At first stages of growth, very small precipitates exhibit coherency hardening as coherency is maintained with the matrix. As the radius increases beyond a threshold, or as full coherency is lost due to a phase transition, the mechanism gradually changes to Orowan strengthening. The maximum strength is achieved at the transition from one mechanism to another, usually occurring at the onset of overaging [36]. Detailed equations for calculating individual strengthening contributions can be found in [5,19,24,35,36].

### 2.2. Experimental Methodology

#### 2.2.1. Material

Material with a composition laying within the specification range of AA6060 alloys was received in the as-cast and homogenized states in the form of 200 mm diameter billets, as well as in the as-extruded and aged states, in the form of a flat, rectangular extrusion profile with a uniform thickness of 1.6 mm.

#### 2.2.2. Characterization

Several specimens for each condition of the material were obtained for characterization and testing. Discs from the middle of as-cast and homogenized billets were cut for easier handling and then 1 cm cube specimens were sampled at ½ of the radius. Rectangular specimens with sides of 1.6 mm × 10 mm × 10 mm were cut from the extruded and aged flat profiles and observed along the extrusion direction. In total, three as-cast, three homogenized, two extruded and two aged specimens were examined, presenting minimal differences between samples of the same condition.

Characterization of the received material included metallography by Light Optical Microscopy (OM) (Leitz Aristomet, Leica Microsystems, Wetzlar, Germany), Scanning Electron Microscopy (SEM), Energy Dispersive X-Ray point analysis (EDX) with associated image analysis and Transmission Electron Microscopy (TEM) analysis.

For metallographic preparation, specimens were subjected to standard grinding and polishing with progressively finer grinding and polishing compounds to achieve a mirror finish. Etching was performed with a Poulton’s reagent consisting of 1 mL HF, 12 mL HCl, 6 mL HNO_3_ and 1 mL H_2_O, modified by the addition of 25 mL HNO_3_ and 12 gr Cr_2_O_3_ (in 40 mL H_2_O) to improve contrast of intermetallics and grain boundaries.

SEM was performed on a Zeiss Ultra 55 LE (ZEISS Microscopy, Jena, Germany) equipped with a Bruker EDX detector. SEM images were acquired in back-scattered electron (BSE) mode. SEM-EDX analysis was performed at 20 kV.

TEM samples of the aged material were prepared using standard twin jet electropolishing and then subjected to TEM analysis on a JEOL JEM-2100 machine (JEOL, Akishima, Tokyo, Japan), operated at 200 kV. Precipitate statistics were measured based on the methodology described by [37].

#### 2.2.3. Tensile Testing

Uniaxial tensile testing of extruded and aged samples was conducted at ambient temperature. The ASTM E8M specification was used to prepare flat tensile specimens, tested in a direction parallel to the extrusion direction. Three specimens for each condition were machined, with an overall length of 180 mm, gage length of 60 mm, width of 12.5 mm and grip width of 20 mm. The thickness was determined by the thickness of the flat extrusion profiles and kept constant at 1.6 mm for all specimens. An INSTRON 8801 servo-hydraulic machine (INSTRON, Norwood, MA, USA) was used to perform tensile testing with a constant crosshead velocity of 0.5 mm/min, whereas the strain was measured with a strain gage extensometer. Note that mechanical properties were measured in a direction parallel to the extrusion direction. It is expected that mechanical properties are different in other directions due to the development of texture upon extrusion. The effect of anisotropic microstructural developments resulting in texture were not considered in the present study and could be the subject of a follow-on work.

## 3. Results and Discussion

### 3.1. Simulation Results

#### 3.1.1. Solidification

**Figure 1:**
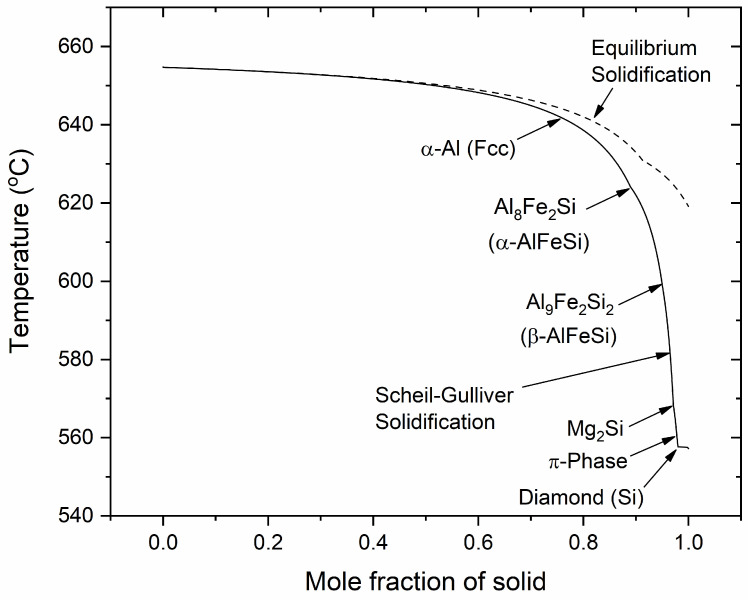
Solidification path predicted with the Scheil-Gulliver model. The dotted line refers to equilibrium solidification.

Solidification behavior during casting was predicted with the Scheil-Gulliver model as presented in Section 2.1.1. The evolution of solid fraction with temperature, and the solidification path solidification is shown in Figure 1 with the solidification sequence predicted to be:

α-Al (FCC) → α-AlFeSi → β-AlFeSi → β-Mg_2_Si → π-phase → diamond (Si)

Solidification begins at the equilibrium liquidus temperature of $T_{lq}=655\;℃$, when α-Al (FCC) dendrites nucleate and grow from the liquid. At approximately 62 °C, the α-AlFeSi (Al_8_Fe_2_Si) starts solidifying along α-Al. Growth of the α-AlFeSi is hindered at approximately 598 °C, when β-AlFeSi (Al_9_Fe_2_Si_2_) nucleates and grows with α-Al, until the end of solidification. At the later stages of solidification, β-Mg_2_Si and π-phase (Al_18_Fe_2_Mg_7_Si_10_) begin to grow at 568 °C and 560 °C, respectively, along with the β-AlFeSi and the α-Al. At 557 °C, diamond (Si) appears and solidification ends with the formation of a eutectic mixture involving diamond (Si), β-Mg_2_Si, π-phase, β-AlFeSi and α-Al, at $T_{sd}=557\;℃$ which is the non-equilibrium solidus temperature. It is noted that the π-phase can be found in eutectic mixtures with diamond (Si) as well as in isolated particles adjacent to β-AlFeSi, in line with the SEM-EDX experimental observations presented in later sections of the work.

#### 3.1.2. Solidification Cooling and Homogenization

**Figure 2:**
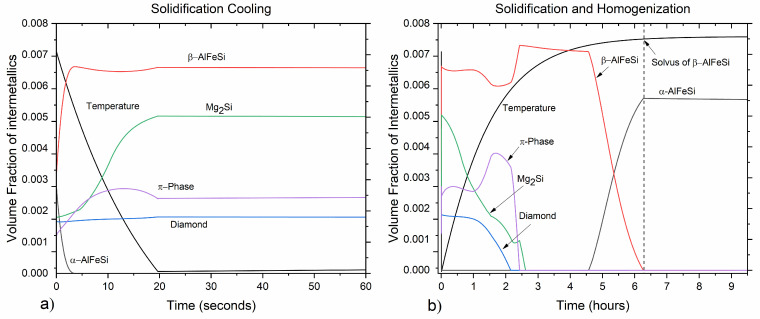
Diffusion calculation results showing phase fractions evolution during (**a**) solidification cooling and (**b**) homogenization.

**Figure 3:**
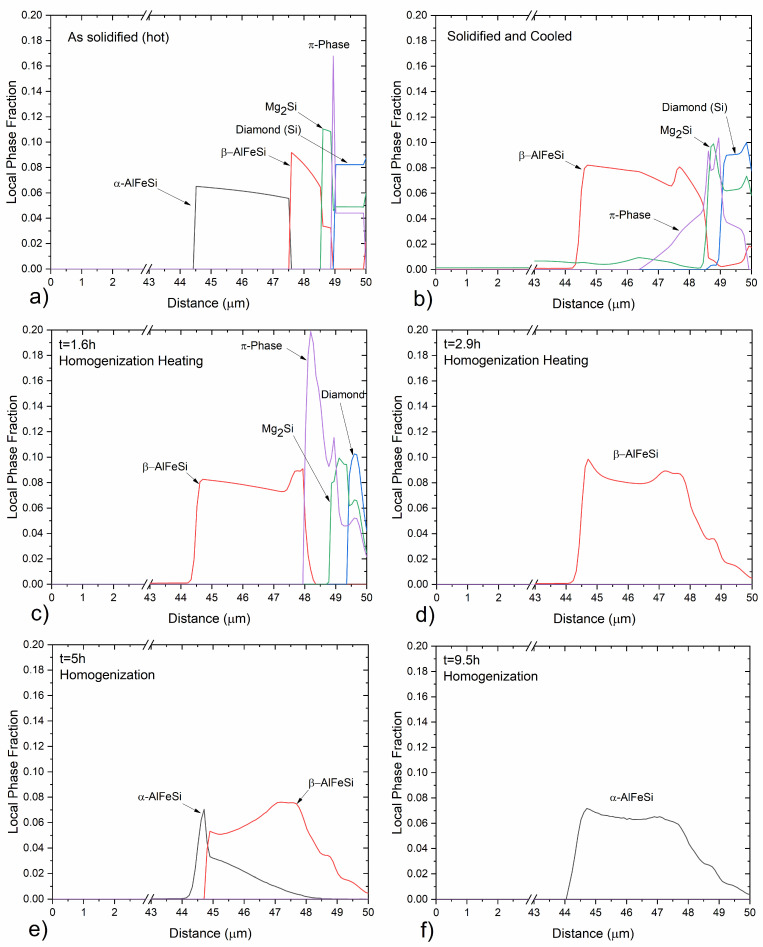
Predicted local fraction profiles of intermetallic phases in the diffusion cell at selected times during (**a**) solidification, (**b**)solidification cooling, (**c**,**d**) homogenization heating and (**e**,**f**) holding. A diffusion distance from the center of a primary dendrite arm to the grain boundary was considered.

**Figure 4:**
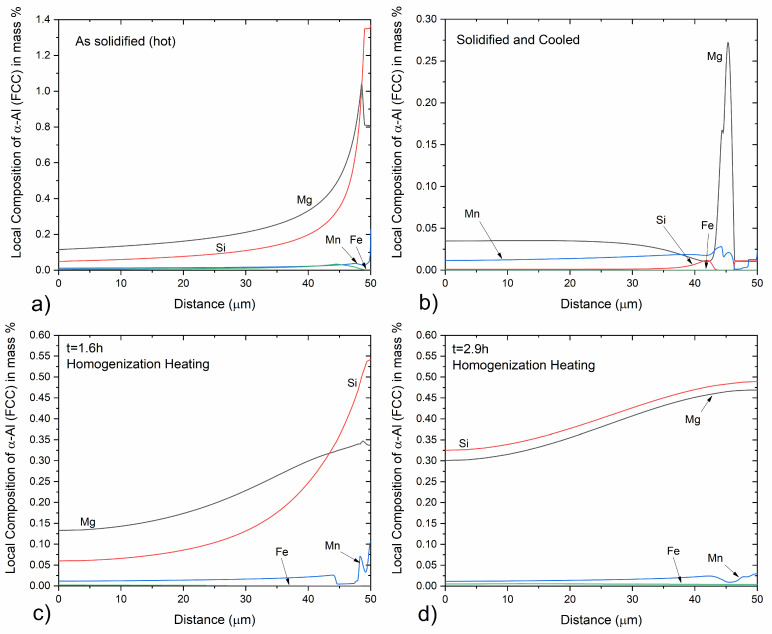
Composition profiles of the α-Al matrix in the diffusion cell, at selected times during (**a**) solidification, (**b**) solidification cooling and (**c**,**d**) homogenization heating. A diffusion distance from the center of a primary dendrite arm to the grain boundary was considered.

The evolution of phase fractions upon cooling after solidification during casting as well as heating and holding during homogenization are presented in Figure 2. Local intermetallics phase fraction profiles in the diffusion cell are shown in Figure 3, whereas composition profiles in the α-Al (FCC) matrix are shown in Figure 4 at selected times during processing. Upon solidification cooling, α-AlFeSi (Al_8_Fe_2_Si) fractions decrease rapidly, dissolving almost completely, whereas β-AlFeSi (Al_9_Fe_2_Si_2_), β-Mg_2_Si, π-phase (Al_18_Fe_2_Mg_7_Si_10_) and diamond (Si) fractions gradually increase. During homogenization, α-AlFeSi nucleates and grows in contact with β-AlFeSi as the β-AlFeSi to α-AlFeSi transformation takes place.

During homogenization heating, the small fraction of α-AlFeSi remaining after solidification cooling, dissolves rapidly while β-AlFeSi, β-Mg_2_Si, π-phase and diamond (Si) gradually decrease in fraction. With an increasing temperature, the eutectic mixture comprised of β-AlFeSi, β-Mg_2_Si, π-phase and diamond (Si) is eliminated, as indicated by the dissolution of diamond (Si). A further increase in temperature results in the gradual dissolution and elimination of the π-phase, followed by the spheroidization and dissolution of β-Mg_2_Si particles. As β-Mg_2_Si, π-phase and diamond (Si) dissolve, Mg and Si are released, and the average composition of the matrix increases. With the increase in temperature, diffusion takes place in the matrix, resulting in the gradual reduction of the elemental segregation developed during casting, as the material homogenizes. Composition profiles at selected times during processing are shown in Figure 4.

After β-Mg_2_Si, π-phase and diamond (Si) have been dissolved, β-AlFeSi is the only intermetallic compound present, as shown in Figure 2b and Figure 3d. Further heating results in only minor morphological changes, with phase fractions remaining almost constant, until the temperature reaches the solvus of α-AlFeSi $T_{Solv}^{\alpha -AlFeSi}=567\;℃$. As the temperature rises above the solvus, α-AlFeSi nucleates adjacent and grows against β-AlFeSi, as the β-AlFeSi to α-AlFeSi transformation initiates, shown in Figure 2b and Figure 3e,f. The transformation presents an exact spatial correlation as α-AlFeSi particles grow on-top of preexisting β-AlFeSi particles. Since the heating rate is relatively low and diffusion is accelerated due to the elevated temperature (above 500 °C), the β-AlFeSi to α-AlFeSi transformation is limited by temperature increase and not time. The diffusional processes contributing to the transformation are significantly faster compared to the heating rate, resulting in the establishment of near equilibrium conditions for a given temperature. The model prediction is in agreement with SEM-EDX measurements discussed below, indicating completion of the β-AlFeSi to α-AlFeSi transformation and elimination of segregation by the end of homogenization at 580 °C.

Upon homogenization cooling, β-Mg_2_Si or β′ can precipitate in bulk or preferentially on dispersoids, α-AlFeSi or grain boundaries. Such precipitates are typically fine and can redissolve rapidly upon extrusion cooling, resulting in a microstructure very similar to the homogenized condition prior to cooling. Large precipitates that could remain undissolved were not observed in the homogenized material via SEM-EDX, with the exception of very few coarse β-Mg_2_Si particles persisting from solidification. Therefore, homogenization cooling was safely omitted in the calculations and the composition of the homogenized material was used for precipitation calculations.

#### 3.1.3. Simulation of Precipitation during Extrusion Cooling

**Figure 5:**
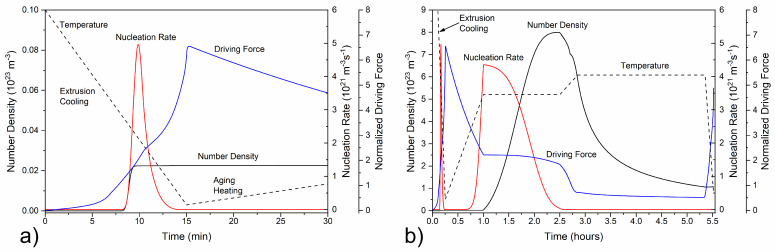
Nucleation rate, number density, and normalized driving force for β″ precipitation upon (**a**) extrusion cooling and (**b**) aging.

**Figure 6:**
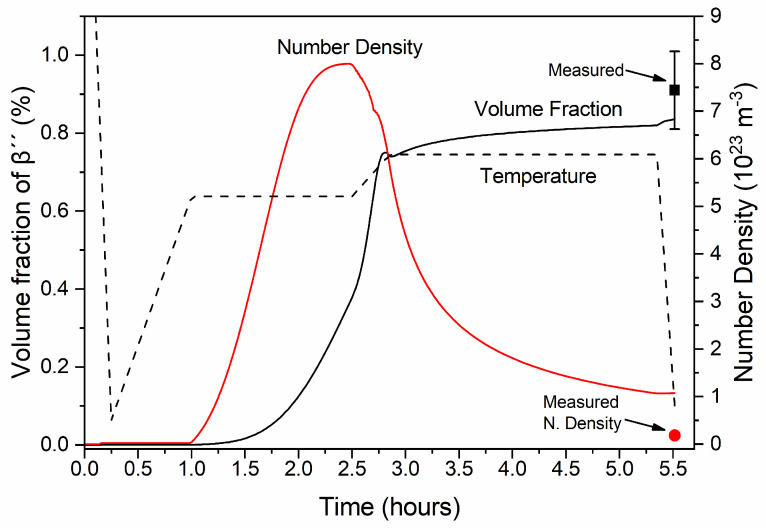
Volume fraction and number density of β″ needles during extrusion cooling and aging. Measurements, with associated standard error bars at the end of aging, validate model predictions.

**Figure 7:**
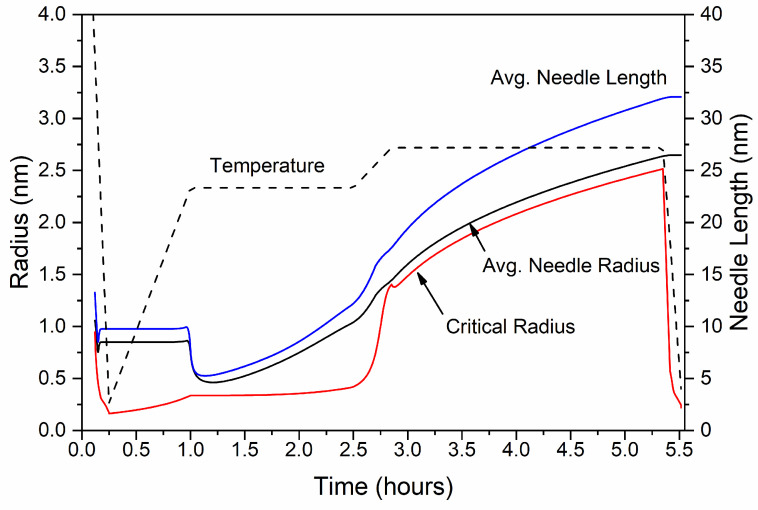
Mean length, mean radius, and critical radius for nucleation of β″ needle-shaped precipitates during extrusion cooling and aging.

**Figure 8:**
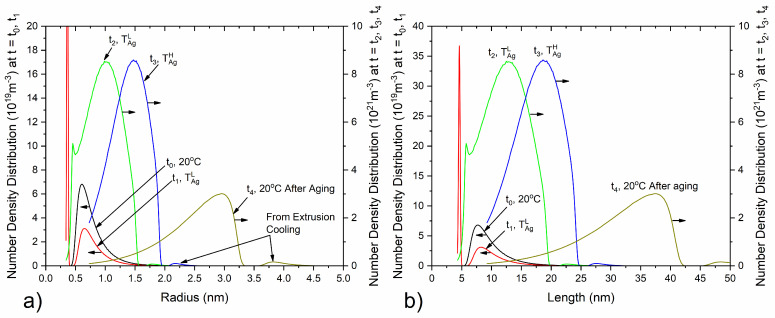
Distribution of β″ (**a**) radius and (**b**) length at different stages of processing. The left scale refers to distributions after extrusion cooling ($t_{0}$) and at the beginning of first aging step ($t_{1}$) at $T_{Ag}^{L}$. The right scale refers to distributions at the end of the first aging step ($t_{2}$, $T_{Ag}^{L}$), the beginning ($t_{3}$, $T_{Ag}^{L}$) and the end ($t_{4}$, $T_{Ag}^{H}$) of the second aging treatment.

**Figure 9:**
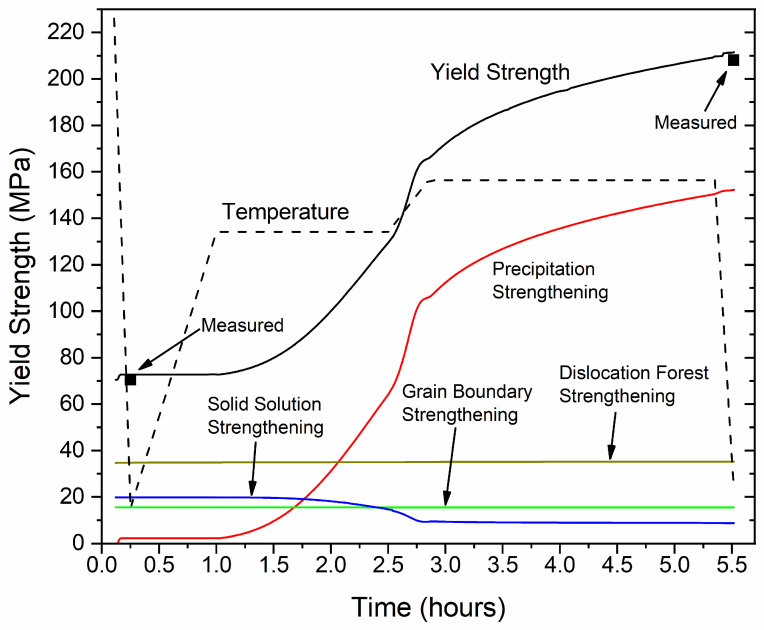
Evolution of total yield strength and individual strengthening contributions with aging time. Yield strength measurements, before and after aging, validate model predictions.

Precipitation simulations of metastable β″ needle-shaped particles upon extrusion cooling and aging were caried out with TC-Prisma in addition to room temperature yield strength calculated. Simulation results are presented in Figure 5, Figure 6, Figure 7, Figure 8 and Figure 9 along with the available measurements for validation. Upon extrusion, the material can exceed the maximum homogenization temperature, reaching temperatures of up to 600 °C, due to adiabatic heating. Yet β-Mg_2_Si-type phases become thermodynamically stable at much lower temperatures. To reduce computational overhead, precipitation simulations for extrusion cooling started at 500 °C.

The number of particles per unit volume, i.e., the number density, the rate of β″ nucleation and the normalized driving force for precipitation of β″ needles during extrusion cooling are presented in Figure 5a, where a constant cooling rate was used for the calculations. Note that driving force is normalized by the factor RT [J/mol], with R being the ideal gas constant and T the temperature in K. At elevated temperatures after extrusion, β″-Mg_5_Al_2_Si_4_ is thermodynamically unstable and the driving force for precipitation is zero. With the temperature decrease, β″-Mg_5_Al_2_Si_4_ becomes stable and the driving force increases as the supersaturation of Mg and Si in the matrix becomes larger. However, nucleation is hindered as the interfacial and strain energy dominate over the chemical driving force for precipitation. As the temperature decreases further, the chemical driving force dominates, promoting the nucleation of β″. The nucleation rate increases rapidly and many β″ particles form raising the number density. The nucleation rate peaks and then gradually decreases, reaching a very small constant value, designating the end of the first nucleation event. Accordingly, the number density rapidly increases, reaching a plateau as new particles stop precipitating at low temperatures. This is due to the stagnation of diffusional processes as the temperature decreases, hindering nucleation of β″ particles. Additionally, as diffusion is limited at low temperatures below ~100 °C, the particles do not grow significantly, resulting in a small volume fraction and number density after extrusion cooling, as shown in Figure 6. The evolution of the critical and mean particle radius, along with the average needle length is shown in Figure 7. Note that the computed ellipsoidal length has been converted to the equivalent rhomboidal needle length so that calculation results are directly comparable with experimental measurements, as described in Section 2.1.4.

#### 3.1.4. Simulation of Precipitation during Aging

Double aging with isothermal holding at a low aging temperature $T_{Ag}^{L}$ (165–180 °C) and a high aging temperature $T_{Ag}^{H}$ (195–210 °C) follows extrusion cooling, to harden the material by precipitating a fine dispersion of β″ needle-shaped particles, with simulation results shown in Figure 5, Figure 6, Figure 7 and Figure 8. Natural aging in the time between extrusion cooling and artificial aging was deemed negligible as described in Section 2.1.4, and calculations were therefore omitted. As the temperature slowly rises to reach $T_{Ag}^{L}$, the precipitation driving force is relatively high, yet due to sluggish diffusion below ~100 °C, nucleation and growth of precipitates is inhibited, with β″ fractions remaining small and the mean radius constant. As $T_{Ag}^{L}$ is approached, the nucleation rate of the β″ particles rises, reaching a maximum value as the temperature becomes high enough to overcome the activation energy for nucleation, and a second nucleation event takes place, yet phase fractions and number densities still remain low. The radius and length distribution of β″ at the end of the first heating stage is shown in Figure 8, as converted from the computational ellipsoidal to the physical rhomboidal geometry. The distribution means are shifted towards higher values, due to particle growth, resulting in a marginally higher mean radius and length, as compared to the end of extrusion cooling (20 ℃).

At the beginning of isothermal holding at $T_{Ag}^{L}$, the nucleation rate of β″ reaches a peak resulting in rapid nucleate of new precipitates, gradually decreasing as nucleation sites become occupied and the precipitation driving force is reduced due to the decreasing supersaturation of Mg and Si in the matrix. As a result, the number density rises, reaching a maximum at the end of the first isothermal holding, whereas the volume fraction of β″ needles increases steadily as a result of the combined effects of nucleation and growth. Yet particles remain small at the end of the first aging step, with the critical radius, the mean radius and needle length continuously increasing but taking very small values in the order of 1 nm. Examination of the particle size distributions indicates that a multi-modal distribution has formed at the end of the first isothermal holding ($t_{2},\;T_{Ag}^{L}$), with a peak located at approximately r = 0.4 nm, a second broader one at r = 1 nm, and a very small peak at r = 1.8 nm. The first peak corresponds to new precipitates nucleating with the critical radius $r_{c}$, whereas the second peak is formed due to the growth of nucleated precipitates during isothermal holding. The third small peak is due to growth of β″ that nucleated during extrusion cooling and it is more pronounced in further stages of processing, as radius and length increase further.

Heating to $T_{Ag}^{H}$ with a constant heating rate and isothermal holding follows. During this stage, very few new precipitates nucleate, with the nucleation rate being negligible. The reduced supersaturation of Si and Mg in the Al matrix as β″ grows, in addition to the increased temperature, results in a reduction of the nucleation driving force. Growth and coarsening of β″ needles are promoted instead of nucleation, resulting in a small increase in volume fraction and a substantial decrease in number density. Small particles dissolve whereas larger ones grow preferentially, driven by the minimization of surface energy. As a result, the needle length and radius distributions shift to higher values and the average radius and length increase.

Rapid cooling to ambient temperature follows after the second isothermal holding, with a marginal increase in volume fraction as nucleation is inhibited due to the high cooling rate. At the end of processing, 0.837% of β″ is predicted, very close to the experimentally determined phase fraction of 0.91% with TEM measurements. A detailed comparison of the experimental and numerical results after extrusion cooling and aging is presented in Section 3.2.5 and Section 3.2.6. The multi-modal size distribution of β″ formed during aging is maintained upon cooling to room temperature, where a broad skewed distribution is observed, with a mean value of 2.65 nm and 32 nm, respectively. Note that the secondary peaks in the radius and length distributions, located approximately at 3.8 nm and 48 nm in Figure 8, originate from the growth of precipitates that formed upon extrusion cooling. Overaging precipitates such as β′, U1, U2 or B′ are expected in this peak, however, in the present study, only precipitation of β″ was considered for extrusion cooling.

#### 3.1.5. Yield Strength Evolution during Aging

The evolution of the room temperature yield strength upon processing, including cooling after extrusion and subsequent aging is presented in Figure 9. The total yield strength, along with the individual contributions due to precipitation hardening, dislocation forest hardening, grain boundary and solid solution are presented and compared against experimental yield strength measurements. Note that since the dislocation density and grain diameter change during extrusion cooling and aging is minimal, the respective strengthening contributions were considered constant with values of 35 MPa and 15.5 MPa, respectively. The solid solution contribution evolves during processing, presenting a maximum as the profile exits the extrusion die, when the matrix concentration of Si and Mg peaks. Upon extrusion cooling, a small number of β″ particles form, resulting in the marginal increase in the precipitation strengthening contribution, resulting in a total yield strength of 72.8 MPa at the end of cooling, aligning with the measured yield strength of 70.5 MPa for the as-extruded material. At first stages of aging the yield strength remains constant, until precipitation of very fine β″ needles commences. As β″ forms, the solid solution contribution is reduced, due to the depletion of Si and Mg in the matrix and the precipitation contribution increases gradually during the first isothermal holding at $T_{Ag}^{L}$, accelerating during heating and holding at the following at $T_{Ag}^{H}$, as β″ fractions rise. According to many authors [5,31,35], the critical radius, marking the transition in the precipitation strengthening mechanism from coherency to Orowan hardening, falls within the range of 1.8 to 5 nm, varying with alloy composition and specific strengthening precipitate. In this work, a value of 2.8 nm has been employed. The needles’ mean radius at the end of processing is determined to be 2.65 nm, implying that the predominant precipitation strengthening mechanism is coherency hardening, with a significant portion of particles remaining shearable by dislocations. Yet, both strengthening mechanisms are deemed to be active, indicating that peak hardness has been achieved. The total yield strength of the material follows the behavior of the precipitation hardening contribution, being the major strengthening mechanism upon aging. By the end of aging, a yield strength of 211 MPa is predicted, in good agreement with the measured value of 208 MPa of the aged material. It is noted that UTS was measured at 165 MPa and 230 MPa, with a uniform elongation of 19.7% and 10.1% for the extruded and aged samples, respectively.

### 3.2. Characterization and Experimental Validation

#### 3.2.1. Characterization of the As-Cast Material

**Figure 10:**
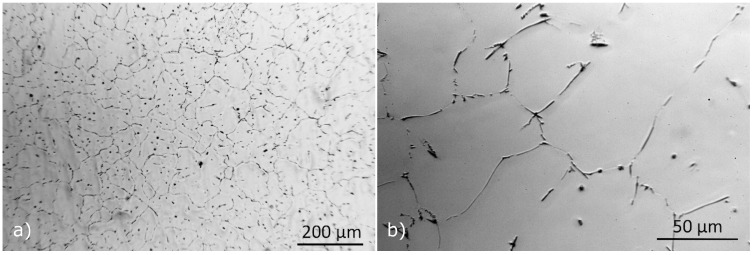
Optical micrograph of as-cast aluminum alloy 6060, at (**a**) ×100 and (**b**) ×500 magnification.

Metallographic images of the as-cast material at 1/2 of the billet radius, using optical microscopy are presented in Figure 10. Intermetallic compounds can be found in the inter-dendritic space and on grain boundaries, with a blocky, acicular or rod-shaped morphology, characteristic of β-AlFeSi (Al_9_Fe_2_Si_2_) particles which are known to limit extrudability [5,30]. Irregularly shaped particles, rounded particles and eutectics were also found to a lesser extent. Intermetallics between secondary dendrites and on grain boundaries indicate the presence of elemental segregation, originating from rapid cooling upon casting. The average grain diameter of the as-cast material was measured at 100 μm, remaining constant even after homogenization.

**Figure 11:**
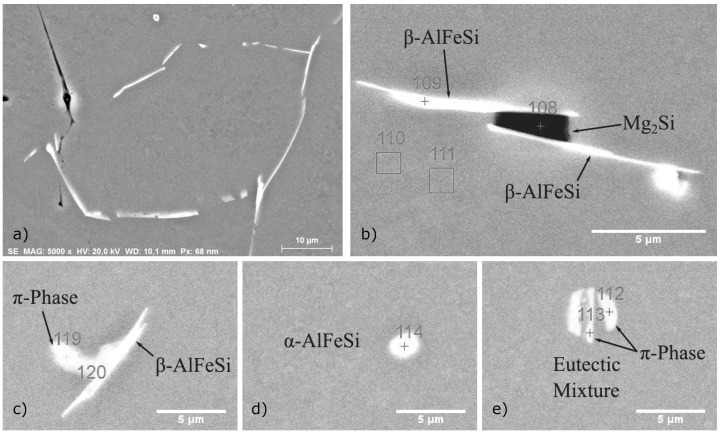
SEM micrographs showing typical microstructural features and intermetallic particles observed in the as-cast material (**a**) near grain boundaries. Blocky β-Mg2Si, acicular and rounded β-AlFeSi, rounded α-AlFeSi, π-phase wetting β-AlFeSi, and eutectic mixtures were observed in (**b**–**e**).

SEM images showing typical microstructural features and intermetallic particles observed in the as-cast condition are presented in Figure 11. Composition analysis of particles with EDX concluded that the majority of particles were coarse β-AlFeSi and β-Mg_2_Si. Yet fewer α-AlFeSi (Al_8_Fe_2_Si), quaternary π-Phase (Al_18_Fe_2_Mg_7_Si_10_) and eutectic particles were also observed. β-Mg_2_Si that formed upon solidification is found in large irregularly shaped or blocky particles that appear dark in the back-scattered SEM micrographs (see Figure 11b). Fine dispersions of β-Mg_2_Si might also be present after solidification cooling, though could not be resolved using SEM-EDX. Intermetallics other than β-Mg_2_Si appear bright in the SEM images, the majority of which were identified as β-AlFeSi, found near grain boundaries with an elongated, acicular morphology. Smaller, rounded particles were also found, having a β-AlFeSi or an α-AlFeSi-phase structure (see Figure 11d). The quaternary π-Phase was found as distinct particles adjacent to (wetting) β-AlFeSi or in the form of eutectic mixtures, presenting a characteristic lamellar morphology (see Figure 11e). Eutectics formed at later stages of solidification with the remaining liquid solidified isothermally as predicted in Scheil simulations. The phases included in the eutectic mixture include the quaternary π-Phase, β-Mg_2_Si, β-AlFeSi and diamond (Si) along with α-Al (FCC) aluminum matrix. It should be noted that in many cases EDX can give inconsistent chemical compositions of phases, especially for small particles. XRD can be a more reliable method for identifying and measuring the fraction of phases present in sufficient quantities after casting or homogenization, however the method was not available to the authors at the time of the study.

#### 3.2.2. Characterization of the As-Homogenized Material

**Figure 12:**
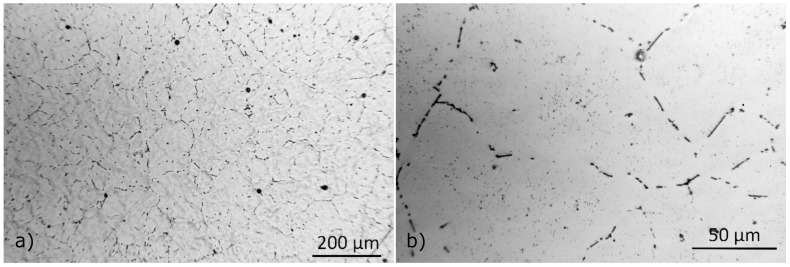
Optical micrograph of the homogenized aluminum alloy 6060, at (**a**) ×100 and (**b**) ×500 magnification.

Metallographic images of the as-homogenized material at 1/2 of the billet radius, using optical microscopy are presented in Figure 12. The average grain diameter was measured at 100 μm, similar to the as-cast grain diameter. Metallographic observations reveal that dendritic structures, observed in the as-cast material, have been eliminated during homogenization, indicating that elemental segregation was removed to a great extent. This was also confirmed by EDX analysis discussed below. No eutectic particles were observed, and the Fe-bearing intermetallics found presented morphological changes typical in homogenized materials including rounding of particle edges, pinching of elongated particles and segmentation into smaller particles to a lesser extent [30].

**Figure 13:**
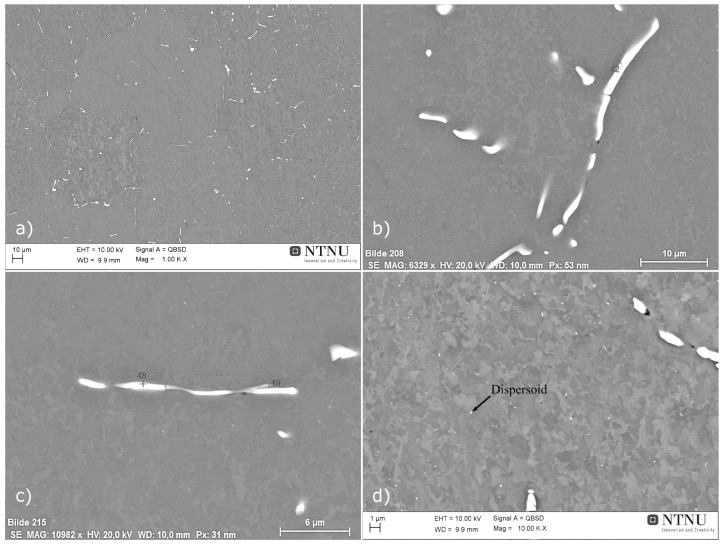
SEM micrographs showing typical microstructural features and intermetallic particles observed in the homogenized material. Only α-AlFeSi intermetallics were observed in (**a**–**c**), denoting the completion of β- to α-AlFeSi transformation. A small fraction of dispersoids was also observed as shown in (**d**).

SEM micrographs of representative microstructural features in the homogenized material can be found in Figure 13. In contrast to the as-cast condition, the homogenized material presents a reduced fraction of intermetallic particles. Using EDX local composition measurements, it is concluded that the predominant intermetallic phase remaining after homogenization is α-AlFeSi (Al_8_Fe_2_Si), with a minimal fraction of blocky β-Mg_2_Si also remaining undissolved. No β-AlFeSi (Al_9_Fe_2_Si_2_), π-Phase (Al_18_Fe_2_Mg_7_Si_10_) or eutectic particles were observed, indicating that the β-AlFeSi to α-AlFeSi has been completed and the as-cast morphology, accompanied by elemental segregation, has been eliminated. Most of the α-AlFeSi particles appear as high aspect ratio, acicular particles with rounded edges, pinched along their length. In comparison to β-AlFeSi particles in the as-cast material, α-AlFeSi particles in the homogenized material appear smaller, with a lower aspect ratio. However, α-AlFeSi, has not been fully spheroidized yet. Additionally, dispersoids were found within grains and along grain boundaries, presented in Figure 13d. The size and fraction of the dispersoids was too small to quantify via SEM-EDX measurements. Presumably consisting of the Mn and Si rich α-Mn (Al_15_Si_2_M_4_) phase which is similar in structure to α-AlFeSi, dispersoids often form upon homogenization of Mn containing 6xxx aluminum alloys. Thermodynamic calculations at the homogenization temperature, including the α-Mn phase supported this hypothesis.

#### 3.2.3. Experimental Validation of Solidification and Homogenization Simulations

EDX measurements were used to identify the phase of intermetallics observed in the material, and image analysis of SEM micrographs was employed to measure their fraction. In the as-cast condition, α-AlFeSi (Al_8_Fe_2_Si), β-AlFeSi (Al_9_Fe_2_Si_2_), π-phase (Al_18_Fe_2_Mg_7_Si_10_), eutectics and β-Mg_2_Si particles were identified. As discussed in Section 3.2.1, blocky β-Mg_2_Si particles appeared dark in the BSE-SEM images, whereas α-AlFeSi, β-AlFeSi, π-phase and eutectics appeared bright, which enabled measurements of their area fraction via image analysis. Due to their low fraction, it was challenging to quantify the fraction of π-phase, α-AlFeSi and eutectic particles, separately from β-AlFeSi. Therefore, the collective volume fraction of Fe-bearing intermetallics was measured via image analysis of SEM micrographs. Blocky β-Mg_2_Si, formed during solidification were measured separately, since they could easily be resolved in the SEM micrographs. Additional β-Mg_2_Si can be found within eutectic particles, though since the resulting eutectic structure was very fine, it was not possible to quantify using SEM-EDX. XRD could have provided supplementary measurements for validation of phase fraction predictions, however it was not available at the time of the study. EDS phase fraction measurements after solidification are compared against model predictions in Table 1. Volume fractions of 0.662% β-AlFeSi, 0% α-AlFeSi, 0.234% π-phase and 0.38% eutectic mixtures are predicted at room temperature, after solidification and cooling. Considering that β-AlFeSi and π-phase are also found within eutectic particles, and subtracting this contribution so that is not accounted twice in the sum, gives a total of 1.199% of Fe-bearing intermetallics, in agreement with the measured fraction of 1.2 ± 0.1%. According to model predictions, the fraction of β-Mg_2_Si trapped within the eutectic mixture is 0.128% and 0.291% could be precipitated in the matrix during cooling, though this was not possible to verify using SEM-EDX. Removing the fraction contributing to eutectics from the total predicted β-Mg_2_Si of 0.506% after solidification gives the pre-eutectic fraction of blocky β-Mg_2_Si at 0.087%. The measured fraction was somewhat lower at 0.017 ± 0.003%, though this small difference can be due to a limited sample size. Solidification model predictions present excellent agreement with measurements in the as-cast condition, accurately predicting the fractions of eutectic, π-Phase, β-AlFeSi and β-Mg_2_Si particles. Note that to decouple the fraction of blocky β-Mg_2_Si from the total β-Mg_2_Si fraction, the local phase fraction profiles of Figure 3 were used. Overlapping phases in the diffusion cell of Figure 3 correspond to intermetallic mixtures such as quaternary eutectics. Integrating over the overlapping regions allows for the calculation of intermetallic mixture fractions and the isolation of blocky β-Mg_2_Si fractions, so that predictions are directly comparable with experimental measurements. The approach has been applied successfully to different 6xxx Al alloys as presented in [5]. In the case that individual phase fraction measurements are available, e.g., from XRD analysis, numerical predictions could be directly compared against experimental values without any conversion, providing a more concrete validation.

In the homogenized material, α-AlFeSi (Al_8_Fe_2_Si) and blocky, pre-eutectic β-Mg_2_Si particles were observed with SEM-EDX suggesting that the β- to α-AlFeSi transformation has been completed and the cast microstructure has been eliminated with the complete dissolution of π-phase and eutectics. A comparison between measured and predicted phase fractions after homogenization is shown in Table 2. The volume fraction of α-AlFeSi was determined to be 0.6%, while that of β-Mg_2_Si particles measured at 0.026%, slightly elevated compared to the as-cast material, attributed to experimental uncertainties. Fine precipitates of β-Mg_2_Si or metastable β′ or β″ could also be present after homogenization cooling, though were not possible to resolve using SEM-EDX due to their small size. According to homogenization simulations, the fraction of α-AlFeSi is 0.554% and that of β-AlFeSi is 0%, agreeing with experimental observations indicating that the β- to α-AlFeSi transformation is complete. Dissolution of eutectic mixtures, β-Mg_2_Si and π-phase particles is also accurately predicted, with a small discrepancy regarding the fraction of β-Mg_2_Si attributed to variations in the particle size, requiring longer heat treatment times to dissolve very large particles. The predicted phase fractions are in alignment with the experimental measurements, validating the modeling approach. Note that dispersoids were also found after homogenization cooling, however since it was not possible to quantify using SEM due to their small size and fraction, were omitted in the calculations in the interest of simplicity. In the present study, measurements were performed only at the end of solidification cooling and homogenization. Yet the accuracy of similar diffusion simulation setups in 6xxx Al alloys has been validated experimentally with XRD, EDS and SEM measurements by the authors [5,11]. XRD was not available in the present study, yet SEM-EDS measurements aligned very well with model predictions for both the as-cast and homogenized materials.

#### 3.2.4. Characterization of the as-Extruded Material

SEM-EDX analysis was employed to characterize the microstructure of the material in the as-extruded condition, aiming at identifying the phases and morphology after extrusion and subsequent cooling. The growth of β-Mg_2_Si-type precipitates on Fe-bearing intermetallics such as α-AlFeSi (Al_8_Fe_2_Si) and α-Mn (Al_15_Si_2_M_4_) dispersoids was investigated since it can promote the development of Precipitate-Free Zones (PFZ) during aging. Although it is expected that due to severe deformation the grain size after extrusion is smaller compared to that of the as-cast and homogenized conditions, grain size measured were not performed, with emphasis placed on studying the intermetallics present after extrusion cooling.

**Figure 14:**
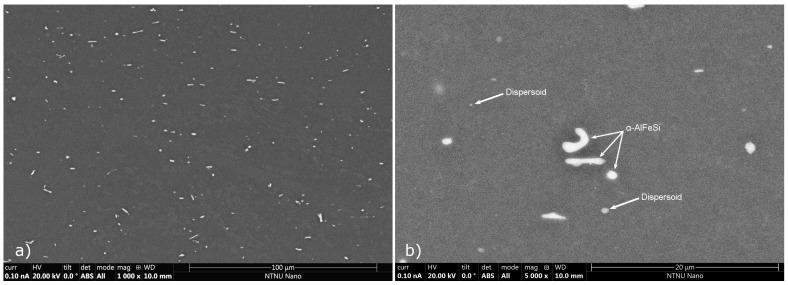
SEM micrographs of typical microstructural features in the as-extruded material at (**a**) ×1000 and (**b**) ×5000 magnification, indicating the presence of only α-AlFeSi as rounded particles and dispersoids.

A typical microstructure of the as-extruded material is presented in Figure 14, using Back Scattered Electron (BSE) SEM, with bright particles corresponding to Fe-bearing intermetallics that remained from homogenization. In accordance with observations for the as-homogenized material, after extrusion cooling only α-AlFeSi and α-Mn (Al_15_Si_2_M_4_) dispersoids were present. α-AlFeSi particles appear smaller and more rounded, with an aspect ratio close to one and smooth edges compared to the as-homogenized material, presumably due to reheating and severe deformation during extrusion, promoting segmentation and spheroidization of large, elongated particles.

β-Mg_2_Si intermetallics were not observed with SEM in the as-extruded material, indicating that the small fractions of blocky β-Mg_2_Si remaining after homogenization, were completely dissolved upon extrusion. Newly formed, coarse β-Mg_2_Si particles on dispersoids, α-AlFeSi or grain boundaries were not observed either. EDX measurements of local composition confirmed that in the matrix, the average Mg concentration was near the alloy’s nominal concentration. In contrast, the matrix concentration of Si was lower than the nominal due to the presence α-AlFeSi and α-Mn (Al_15_Si_2_M_4_) dispersoids, as expected. Although coarse Mg-Si precipitates were not observed, it expected that very fine precipitates of phases such as β-Mg_2_Si, β″, β′, B′, U1 or U2, nucleated on α-AlFeSi, dispersoids and grain boundaries after extrusion though with a very low volume fraction, as suggested by precipitation model predictions. Those nuclei grow during aging and contribute to the formation of PFZ, and were studied in the aged material using TEM, discussed in the following section.

#### 3.2.5. Characterization of the Aged Material

Transmission Electron Microscopy was employed to study the material following extrusion and aging. The aim was to analyze the structure and morphology of the strengthening precipitates, investigate the occurrence of overaging particles growing on grain boundaries, α-AlFeSi particles or dispersoids, contributing to the development of Precipitate-Free Zones (PFZs). Upon aging, it is expected that the grain structure remains unchanged, however grain size measured were not performed in the aged condition, with emphasis placed on studying strengthening precipitates and PFZs.

**Figure 15:**
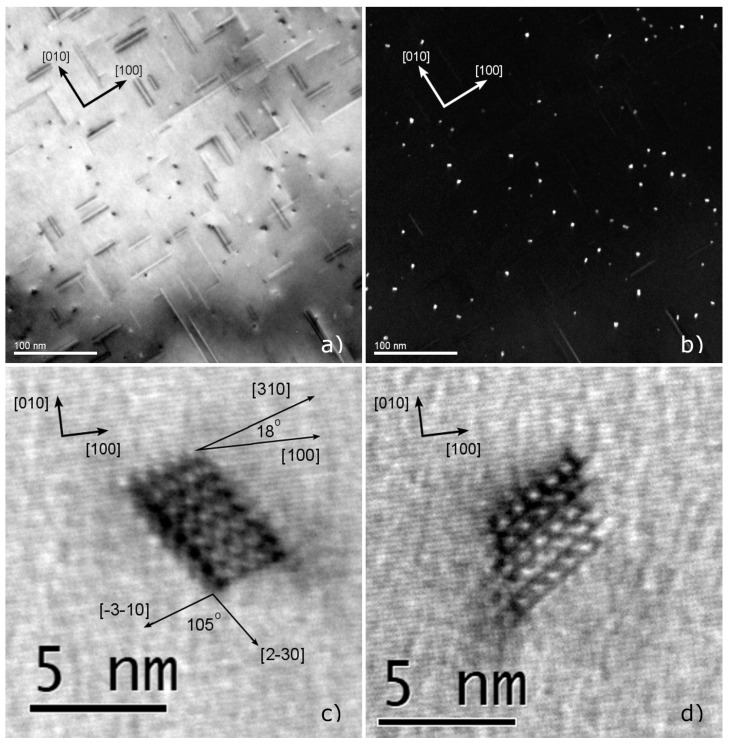
Typical TEM micrographs of the aged material along <001>Al. Strengthening precipitates are identified as β″. Bright and dark field images are shown in (**a**,**b**) whereas cross-sections β″ in high magnification are shown in (**c**,**d**).

Typical TEM micrographs are shown in the bright and dark field images of Figure 15a,b, with a cross-section of precipitates in high magnification presented in Figure 15c,d. As evidently shown in the figure, the needle-shaped precipitates grow preferentially along the three <100>Al directions, and as such the aged material was examined along a <100>Al direction. This aligns with crystallographic orientation of β″ [33,38,39,40], suggesting that β″ is the strengthening precipitate observed. Further observations of the morphology and orientation of cross-sections confirmed that the observed particles corresponded to the metastable β″-Mg_5_Al_2_Si_4_. More specifically a monoclinic unit cell with sides a=15.16 Å, b=4.05 Å, c=6.74 Å, angle $\hat{\beta }=105.3{}^{\circ}$, and a unit cell volume of $V_{cell}=399.2\;{Å}^{3}$, characteristic of β″-Mg_5_Al_2_Si_4_ was observed as shown in Figure 15c,d. It can be considered as a super-cell in the Al matrix with a||<320>Al, b||<001>Al and c||<310>Al. Consequently, β″-Mg_5_Al_2_Si_4_ needle-shaped particles maintain fully coherency with the aluminum matrix on all sides and the lowest interfacial energy is observed on the {130}Al and {320}Al planes, along the <001>Al needle directions [33,38]. The crystallographic relations of the needles with the aluminum matrix are presented in Figure 15c, where the angle between the small particle side, parallel to the [310] direction and the [100] direction of the Al matrix, was measured at 18°. Additionally, the angle between the $\left[\bar{3}\bar{1}0\right]$Al and the $\left[2\bar{3}0\right]$Al directions which are parallel to the sides of the particle, was measured at 105°, reflecting the monoclinic angle $\hat{\beta }=105.3{}^{\circ}$ of the unit cell and confirming that indeed the needles are comprised of the β″-Mg_5_Al_2_Si_4_ phase.

**Figure 16:**
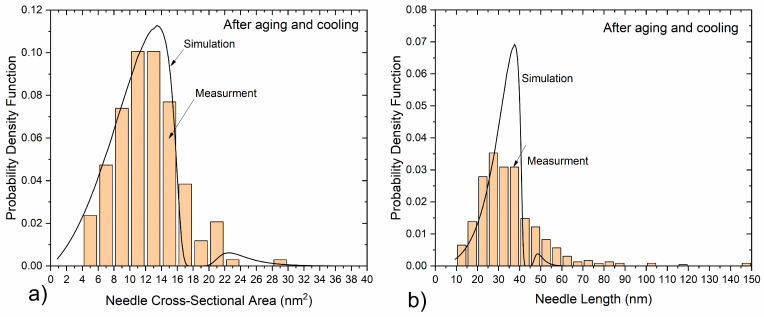
Predicted and measured distributions of β″ needle (**a**) cross-sectional area and (**b**) length after aging.

The size distribution of β″-Mg_5_Al_2_Si_4_ particles, i.e., the cross-sectional area and length distributions of needles was determined experimentally utilizing image analysis of bright field TEM micrographs similar to that of Figure 15a. Dark field TEM micrographs were employed to determine the average count of precipitates per unit volume, i.e., the average number density since they provided better contrast for the needle cross-sections. Additionally, the two-beam CBED technique used for measuring specimen thickness with high precision required for calculating the number density. To determine the volume fraction β″, the average precipitate volume was multiplied with the number density. The measured length and cross-sectional area distributions are shown in Figure 16, as compared to predicted distributions from precipitation simulations converted to the equivalent rhomboidal values as explained in Section 2.1.4. Measured needles range in cross-sectional area from 4 to 30 nm^2^, with an average value of $\bar{A}$ = 12.3 nm^2^. The distribution is skewed towards smaller particles as very few needles with a cross-section larger than 20 nm^2^ were detected. Accordingly, the measured needle length distribution is also skewed towards shorter particles, ranging from 10 to 240 nm, with an average length of $\bar{L}$ = 40 nm.

Considering that β″ needles have a rhomboidal cross-section, comparing precipitation model predictions with quantitative TEM measurements requires the conversion of area measurements by defining an equivalent radius. As elaborated in Section 2.1.4, the maximum rhomboidal radius $r_{rm}$ can be calculated from the cross-sectional area measured and then directly compared against precipitation model predictions as $r_{rm}=\sqrt{\frac{1+\cos\left(\theta \right)}{2\;sin(\theta )}\;A}$. With a value of of θ = 75°, originating from the monoclinic unit cell angle, and a mean area value of $\bar{A}$ = 12.3 nm^2^, from measurements, the average maximum rhomboidal radius is calculated as ${\bar{r}}_{rm}$ = 2.83 nm. The average aspect ratio of β″ needles was estimated based on the average length and equivalent radius as $AR=\bar{L}/(2{\bar{r}}_{rm})=7.06$, with $\bar{L}$ = 40 nm as measured with TEM. Note that in order to compare length measurements with model predictions a volume correction factor needs to be applied as explained in Section 2.1.4. The number density of β″ precipitates, i.e., the number of particles per unit volume, was measured at N = 1.85·10^22^ m^−3^ and the volume fraction at $f_{\beta \prime \prime }$ = 0.91%. A summary of measurements is presented in Table 3.

The properties of β″ particles as measured via quantitative TEM analysis indicates that the full potential for precipitation of metastable strengthening precipitates has been achieved, as a result of aging processing conditions. The volume fraction is near the equilibrium value of β″ and the Si and Mg supersaturation in the aluminum matrix has been eliminated. A fine dispersion of particles has been achieved with a high number density due to the elevated driving for nucleation, promoting nucleation during the first aging treatment. Growth to reach near equilibrium fractions took place during the second aging treatment, without resulting in coarsening and overaging. The β″ particles maintain full coherency with the matrix and no overaging precipitates such as β′, U1, U2, B′ or β-Mg_2_Si were found in the bulk. Despite the increased needle length, the radius is very small and particles remain shearable by dislocations, contributing to strengthening via coherency hardening. As discussed in Section 3.1.5, transition to the Orowan strengthening mechanisms for β″ takes place at the critical radius of 2.8 nm, which is very close to the measured radius of ${\bar{r}}_{rm}$ = 2.83 nm. This indicates that the aged material is very close to peak hardness, usually achieved at the onset of overaging where transition from coherency to Orowan strengthening takes place and the two mechanisms contribute equally to strength. Overaging precipitates (β′, U1, U2, B′, β-Mg_2_Si) were not found in the bulk of the material, yet they were detected around dispersoids and along grain boundaries. The particles found are associated with development of Precipitate-Free Zones (PFZs) due to slow extrusion cooling as discussed in the following paragraphs and are not an indication of overaging.

**Figure 17:**
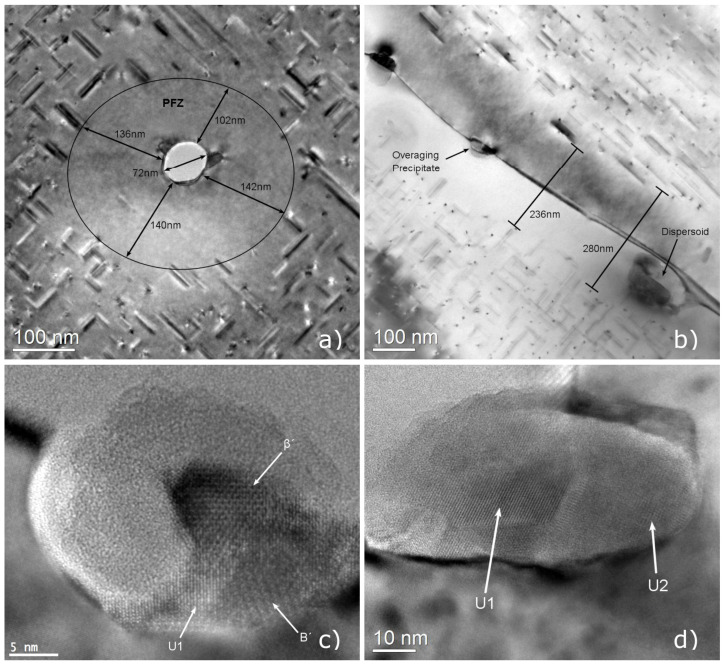
Typical TEM micrographs presenting Precipitate-Free Zones (PFZs) (**a**) around dispersoids and (**b**) along grain boundaries in the aged material. Both dispersoids and grain boundaries were decorated by overaging precipitates, identified as mixtures of β′, U1, U2, B′ and disordered phases, as shown in the high magnification TEM images of (**c**,**d**).

Precipitate-Free Zones (PFZs), where strengthening β″ phase precipitates are missing, were observed around dispersoids and along grain boundaries as shown in the TEM micrographs of Figure 17. Many dispersoids and grain boundaries were also decorated by coarse overaging precipitates, that presumably nucleated upon cooling after extrusion and grew over the course of the aging treatment. The width of PFZs was quantified through bright field TEM micrograph measurements, finding that PFZs around dispersoids varies between 170 and 59 nm, with an average width of 118 nm. Similarly, the average width of PFZs on grain boundaries, was measured at approximately 153 nm. TEM measurements suggested that dispersoids formed during homogenization, were comprised of the Mn and Si rich α-Mn (Al_15_Si_2_M_4_) phase, presenting a spheroidal shape with a diameter ranging from 70 to 140 nm. A large PFZ width is often linked to degraded ductility and uniform elongation after aging. Upon deformation damage, inhomogeneous strain partitioning in PFZs is thought to result in dislocation accumulation and void nucleation leading to premature failure.

The coarse overaging precipitates, decorating grain boundaries and dispersoids can be found in the high-resolution TEM images of Figure 17c,d. The coarse overaging particles have cross-section diameter ranging from 10 to 30 nm, comparable to the diameter of the dispersoids. High-resolution TEM images reveal that those particles include many coprecipitated metastable phases that can be identified, based on their unit cell periodicities. The hexagonal β′ with a unit cell of a=7.15 Å and c=12.15 Å, the trigonal U1 with a unit cell of a=4.05 Å and c=6.74 Å, the orthorhombic U2 with sides a=6.75 Å, b=4.05 Å and c=7.94 Å, and the hexagonal Β′ having a unit cell with sides a=10.4 Å and c=4.05 Å, and disordered precipitates were identified in the overaging precipitate clusters [39,40,41].

The formation of PFZs and overaging precipitates clusters are interlinked, both associated with a low cooling rate after extrusion [16,17,42]. Upon cooling after extrusion, lattice vacancies and solute atoms such as Si and Mg in the matrix migrate towards nearby interfaces, i.e., grain boundaries and dispersoids or α-AlFeSi interfaces, driven by solid state diffusion. Therefore, a region around dispersoids and grain boundaries becomes depleted in vacancies and alloying elements and metastable β-family particles nucleate during cooling preferentially on the interface, where the concentration of solute atoms and vacancies is higher. Upon aging, fine β″ needles precipitate in the bulk, yet not near dispersoids and grain boundaries, where the available nucleation sites are reduced due to a low concentration of vacancies and the driving force for precipitation is decreased due to depletion of Mg and Si, leading to the development of PFZs. Furthermore, solute atoms accumulated near the interface promote the growth of overaging precipitates that were nucleated during cooling, resulting in large precipitate clusters around dispersoids and grain boundaries. The effects are more prominent near grain boundaries, where diffusion is accelerated due to grain boundary diffusion, resulting in increased PFZs.

#### 3.2.6. Experimental Validation of Precipitation Model

To validate the precipitation calculations, model predictions were compared against the available experimental data regarding the number density, volume fraction and particle size distribution of β″, in addition to yield-stress measurements. A summarized comparison between model predictions and experiments is provided in Table 4, where a good agreement is observed. After extrusion, cooling yield strength was measured at 70.5 MPa, very close to the predicted value of 72.8 MPa. In the aged material, the volume fraction and number density of β″ needles was measured at 0.91% and 1.85·10^22^ m^−3^, respectively, aligning well with the predicted values of 0.837% and 1.08·10^23^ m^−3^. The small discrepancy in the number density is linked to an inconsistency in the mean needle length which was measured at 40 nm and predicted at 32 nm. This small discrepancy is attributed to a model limitation, keeping the needle length proportional to the radius via a constant aspect ratio. The distribution tails towards larger needles are under predicted by the model leading to a lower mean length value. Yet the predicted mean needle radius of 2.65 nm was in excellent agreement with the measured value of 2.83 nm. As discussed in Section 2.1.4 and Section 3.2.5, since β″ particles have a rhomboidal cross-section, the maximum rhomboidal radius $r_{rm}$, as calculated from the measured mean cross-sectional area, was used for comparison with simulation results.

Predicted and measured normalized distributions (probability density function PDF) of β″ needle cross-sectional area and length at the end of aging are compared in Figure 16. Equations presented in Section 2.1.4 were used to convert the predicted radius distribution into an area distribution, for direct comparison with experiments. There is an excellent agreement between the predicted and measured radius distributions, with the precipitation model accurately predicting the skewed distribution after aging and the presence of a second small peak, corresponding to particles nucleated during extrusion and grown during aging. The predicted needle length distribution also presents reasonably good agreement with the measured length distribution. The general shape of the distribution is captured well by the model, with minor discrepancies at the distribution tails towards larger needle lengths. The predicted distribution is narrower compared to the observed one, which can be attributed to the constant aspect ratio between needle length and radius used in the model, resulting in small discrepancies in the length and number density. In general, the precipitation model predictions align well with measurements regarding the radius and length distributions, the volume fraction and number density of β″ precipitates. In particular, the volume fraction and radius distribution, which are key parameters influencing strength after aging [35], are in excellent agreement with the experimental measurements.

## 4. Conclusions

In this work, the entire process chain of a 6060 aluminum-alloy, including solidification, homogenization heating and holding, extrusion cooling and aging, was simulated using computational thermodynamic and kinetic modeling. In-depth characterization of the material in the as-cast, as-homogenized, as-extruded and aged conditions was carried out using optical microscopy, quantitative SEM-EDX and high-resolution TEM analysis to observe the relevant microstructural features. From the work presented, the following conclusions can be drawn:

- Solidification and homogenization calculations were in excellent agreement with experimental measurements in the as-cast and homogenized materials. Calculations predict the presence of coarse α-AlFeSi, β-AlFeSi, β-Mg_2_Si, π-phase and eutectics containing diamond (Si) after solidification cooling, agreeing with SEM-EDX measurements. The elimination of the cast structure, the dissolution of β-Mg_2_Si, π-phase and eutectics, and the completion of the β-AlFeSi to α-AlFeSi transformation upon homogenization is also predicted, in accordance with the SEM-EDX observations.
- Regarding the as-extruded material, coarse β-Mg_2_Si particles were not found on dispersoids, grain boundaries or α-AlFeSi particles, after extrusion using SEM-EDX. However, coarse, overaging particles of metastable β-type phases including β′, U1, U2 and B′, were observed on dispersoids and grain boundaries, via TEM in the aged material. The particles nucleated during cooling after extrusion and grew over the course of aging.
- The formation of overaging precipitates near grain boundaries and dispersoids is associated with the development of Precipitate-Free Zones (PFZs), where the strengthening phase β″ does not form upon aging. Extended PFZs were observed in the aged material, resulting from a relatively low extrusion cooling rate.
- In the aged condition, TEM analysis revealed a fine dispersion of needle-shaped precipitates, identified as the fully coherent β″, with a volume fraction 0.91%, a mean radius of 2.8 nm and a mean length of 40 nm.
- Simulations of precipitation kinetics during extrusion cooling and aging predicted the evolution of the volume fraction, number density, radius and length distributions of β″ needles, in excellent agreement with TEM observations. Minor discrepancies regarding precipitate length were observed. The increase in yield strength due to the precipitation of β″ was accurately predicted, with results showing that peak hardness was achieved when the precipitate size was approaching the transition from coherency to Orowan strengthening.
- Precipitation simulations revealed that most β″ precipitates were nucleated during the first aging holding at the low aging temperature $T_{Ag}^{L}$ (165–180 °C), due to a high driving force for precipitation. Yet growth remained slow, due to limited diffusion at low temperatures. During the second isothermal holding at the high aging temperature $T_{Ag}^{H}$ (195–210 °C), the volume fraction increased as particles grow rapidly.
- Simulation predictions presented good agreement with experimental measurements, indicating that the modeling approach can be used to optimize the composition and processing conditions of extrudable 6xxx aluminum alloys.
- With key microstructural features modeled at critical processing stages and calibrated with targeted experiments, the optimization of process parameters is enabled. The approach has the potential of minimizing trial-and-error experiments required to optimize the properties of extrudable Al alloys, reducing time and effort, therefore improving efficiency.

## Figures and Tables

**Table 1:** Model predictions as compared against measured phase fractions in the as-cast material.

	Measured Volume Fraction	Predicted Volume Fraction
β-AlFeSi + α-AlFeSi + π-Phase + Eutectics	1.2 ± 0.1%	1.199%
Blocky β-Mg_2_Si	0.017 ± 0.003%	0.087%

**Table 2:** Model predictions as compared against measured phase fractions in the homogenized material.

	Measured Volume Fraction	Predicted Volume Fraction
α-AlFeSi	0.6%	0.554%
β-AlFeSi	0%	0%
Blocky β-Mg_2_Si	0.026%	0%
π-Phase	0%	0%
Eutectics	0%	0%

**Table 3:** Summary of β″ particle measurements in the aged material.

Measurement	$\bar{A}$ (nm^2^)	$\bar{L}$ (nm)	${\bar{r}}_{rm}$ (nm)	AR	N (m^−3^)	$f_{\beta^{\prime \prime }}$ (%)
**Value**	12.3	40	2.83	7.06	1.85·10^22^	0.91

**Table 4:** Precipitation and strength model predictions as compared against measurements in the as-extruded and aged materials.

	Measurement	Prediction
As-extruded material		
Yield strength (MPa)	70.5	72.8
Aged material		
$\mathrm{Volume}\;\mathrm{fraction}\;\mathrm{of}\;\beta \prime \prime \;f_{\beta \prime \prime }\;$(%)	0.91	0.837
Number density of β″ *N* (m^−3^)	1.85·10^22^	1.08·10^23^
$\mathrm{Mean}\;\mathrm{radius}\;{\bar{r}}_{rm}$ (nm)	2.83	2.65
$\mathrm{Mean}\;\mathrm{length}\;\bar{L}\;$(nm)	40	32
Yield strength (MPa)	208	211

## Data Availability

Data could be made available upon request.
